# MODEL-BASED CLUSTER ANALYSES OF COGNITION FOR UNPACKING SUBGROUP DIFFERENCES IN PSYCHOSOCIAL OUTCOMES

**DOI:** 10.1093/geroni/igz038.2983

**Published:** 2019-11-08

**Authors:** Nelson A Roque, Martin J Sliwinski

**Affiliations:** Pennsylvania State University, Center for Healthy Aging, University Park, Pennsylvania, United States

## Abstract

We forward a methodological approach, using model-based cluster analyses, and ambulatory assessments of cognition (2 indicators from each task), to derive subgroups of interest for tailored clinical follow-up in a longitudinal framework. Community dwelling adults were asked to complete 14 consecutive days of ecological momentary assessments (EMAs) using smartphones, including measures of cognitive performance, and self-reported physical and mental health outcomes (e.g., stress, memory complaints, depression, pain). A stable four-cluster solution emerged, labelled as: (1) a high-risk cognitive group (13%; most memory complaints, slowest performing, more memory errors); (2) subjective risk group (42%; highest levels of somatic and cognitive complaints); (3) normative aging (28%; intermediate cognitive performance -- speed/accuracy); (4) super-cognitive agers (17%; fastest speed, best memory). In conclusion, these findings highlight the potential of a cluster-based approach for risk classification, uncovering different profiles of poor performance that may represent different etiologies.

